# Creating chiral superstructures in magnetic quadrupole fields

**DOI:** 10.1093/nsr/nwad221

**Published:** 2023-09-06

**Authors:** Shu-Hong Yu

**Affiliations:** Department of Chemistry, Hefei National Research Center for Physical Sciences, University of Science and Technology of China, China; Department of Materials Science and Engineering, Southern University of Science and Technology, China

Chiral materials refer to species with chiral symmetry, which means the absence of mirror planes or breaking of inversion symmetry in material structures. A material or structure is chiral if it is distinguishable from its mirror images. That is, the chiral materials and their mirror images do not coincide through rotations or translations. Chiral materials are frequently described as ‘handed’ because our hands themselves are chiral. Chirality exists in nature, including the DNA double helix, proteins, amino acids and human hands. Interestingly, biology only adopts one chirality in nature. While DNA, RNA and their building blocks are all right-handed, amino acids and proteins are all left-handed. The structural homochirality of the fundamental biological molecules is thought to be related to the origin of life but the selectivity remains mysterious [1]. Chirality is also used to describe physical states of matter in quantum mechanics. For example, in addition to linear polarization, in which the electric field of light only oscillates in one plane, circular polarization has a constantly rotated electric field about the light propagation direction. Depending on the spin angular momentum states of photons, circularly polarized light is either left-handed or right-handed.

Chiral materials have many interesting and unique physical properties that originate from chirality. They interact differently with both linearly and circularly polarized light, leading to remarkable optical activity and phenomena. Under excitation of circularly polarized light, optically active materials have circular dichroism because of different absorbance between left-handed and right-handed circularly polarized light. Another interesting property is optical rotatory dispersion under linearly polarized light excitation, resulting in continuous color-switching in a chiral medium.

Driven by these remarkable properties, researchers have developed many methods to create chirality on achiral materials, such as templating methods, mechanical deformation and lithography [2,3]. DNA templates are widely used in this regard to assemble plasmonic nanoparticles into chiral superstructures [4]. Lithography is a top-down fabrication method for preparing chiral structures [5]. However, these existing methods only work for materials of specific shapes, chemical compositions or size ranges. Besides, the chiral structure formation is not reversible. Materials have fixed handedness once made and creating oppositely handed materials requires additional templates or fabrication. These challenges have remained for decades.

**Figure 1.**
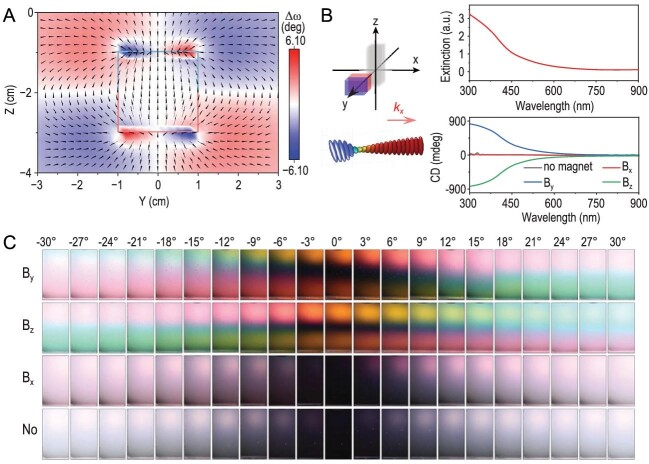
A quadrupole field chirality for creating a chiral superstructure. (A) The field rotating vectors (black arrows) and field angle changes (color map) of the magnetic field are along the *x*-axis. Positive and negative rotation angles (Δω) represent clockwise left-handed and counterclockwise right-handed rotation of the magnetic field, respectively. (B) Absorption spectrum (top) and CD spectra under different magnetic fields (bottom) of Fe_3_O_4_@SiO_2_ nanorods. (C) Digital pictures of the hybrid nanorod dispersion under different magnetic field conditions. The polarization direction (α) of the analyser was switched from −30^o^ to 30^o^ during the measurement. Reprinted with permission from Ref. [6]. Copyright 2023, American Association for the Advancement of Science.

A recent research article in *Science* reports a general assembly approach to chiral superstructures using quadrupole fields of permanent magnets [6]. This new method assembles materials of any size, chemical composition, shape and length scale into chiral superstructures that demonstrate circular dichroism, rotatory dispersion and circularly polarized luminescence by incorporating corresponding guest molecules into magnetically responsive materials. This general method is realized based on the discovery of quadrupole field chirality in the magnetic field of permanent magnets and used for assembling metals, semiconductors, polymers, oxides, small molecules and fluorophores into chiral superstructures, setting the stage for exploiting next-generation materials with highly tunable chirality and optical activity (Fig. 1).

An analytical model has been developed to map the field gradient of permanent magnets, demonstrating quadrupole field chirality. Applying such a chiral magnetic field to magnetic nanoparticles produces chiral superstructures, with handedness and pitch being dependent on magnet positions and field directions. More interestingly, transferring the field chirality to any achiral materials is possible if the guest achiral materials can be properly incorporated into the host magnetic nanoparticles. The authors have shown that chiral superstructures of different types of molecules have been realized by coating or doping guest molecules into the host, including small molecules, dyes, oxides, metals, semiconductors, polymers and fluorophores. They observed CD responses at the characteristic absorbance wavelength of these guest molecules, demonstrating successful transfer of quadrupole field chirality to materials at different length scales. Another advantage of the general method is easy control over structural chirality, leading to dynamic color-switching by changing the magnetic fields.

In short, this general all-scale magnetic assembly adds new toolboxes to create chiral superstructures out of any achiral materials, which produces chiral superstructures in a reversible, contactless and instantaneous way by simply exposing a dispersion of magnetic building blocks to the field of a permanent magnet. It is not limited by the building block sizes, shapes and chemical compositions. The present method will spark research interest in the design of chiral materials of diverse compositions and properties, and enable the creation of novel optical devices featuring smart responses to external stimuli.
